# Idiopathic Granulomatous Lobular Mastitis: An Imitation of Breast Carcinoma

**DOI:** 10.7759/cureus.15206

**Published:** 2021-05-24

**Authors:** Ismail Aydin, Tugrul Kesicioglu, Selahattin Vural, Ilker Sengul, Kivanc Yilmaz, Demet Sengul

**Affiliations:** 1 General Surgery, Giresun University Faculty of Medicine, Giresun, TUR; 2 Endocrine Surgery, General Surgery, Giresun University Faculty of Medicine, Giresun, TUR; 3 Pathology, Ministry of Health-Giresun University Education and Research Hospital, Giresun, TUR; 4 Pathology, Giresun University Faculty of Medicine, Giresun, TUR

**Keywords:** breast cancer pathology, breast, breast neoplasms, granulomatous mastitis, mastitis, mimicking, breast pathology, idiopathic, histopathology (hp), cd68+

## Abstract

Since idiopathic granulomatous lobular mastitis, also known as idiopathic granulomatous mastitis or granulomatous lobulitis, was first described by Kessler and Wolloch in 1972, no consensus on the ideal and definitive treatment for this phenomenon has been reached thus far. Idiopathic granulomatous lobular mastitis mostly frequently observed in women of childbearing age within a few years of pregnancy with a higher incidence in patients of Hispanic, Native American, Middle Eastern, and African descent. This entity, *per se*, is a rare, benign, chronic inflammatory breast condition of unknown aetiology mimicking two common breast disorders. The first is breast inflammations or infection, such as cystic neutrophilic granulomatous mastitis; granulomatous mastitis due to *Corynebacterium*;other infections with granulomas, mycobacteria, fungi, cat scratch disease, and *Brucella*; granulomatosis with polyangiitis (Wegener's granulomatosis); sarcoidosis; and squamous metaplasia of lactiferous ducts. The second is breast carcinoma in some challenging cases. Of note, no consensus, *per se*, has been declared on its therapeutic management. The following vignette case described in this report involves idiopathic granulomatous lobular mastitis imitating breast carcinoma. It is important to note that, the aetiology of idiopathic granulomatous lobular mastitis is unknown, its diagnosis is difficult, and physicians should be vigilant and aware of this condition in order of abstaining from an overtreatment for malignancy or overlooking a true malignancy.

## Introduction

Idiopathic granulomatous lobular mastitis (IGLM), also known as idiopathic granulomatous mastitis or granulomatous lobulitis, was first defined by Kessler and Wolloch in 1972 as a seldom, benign, chronic inflammatory disease of the breast of unknown aetiology mimicking malignancy [1], clinically and radiologically [2]. This remains a diagnostic challenge for clinicians. Furthermore, accurate treatment and management options for this disorder, such as antibiotics, surgical interventions, and corticosteroid therapy, remain controversial. Currently, there is no consensus on the optimal treatment or management protocols for IGLM. Herein, we present a 52-year-old woman with IGLM mimicking carcinoma of the breast [3].

This disorder is characterized by chronic granulomatous inflammation of the lobules without caseous necrosis. IGLM is of unknown origin, and its diagnosis relies on both the demonstration of a characteristic histological pattern and exclusion of other possible causes of granulomatous breast lesions. Clinically and radiographically, IGLM is difficult to differentiate from early-onset breast cancer. The present report describes the clinical features, treatment, and response of an adult who presented with IGLM mimicking malignancy.

## Case presentation

**Figure 1:**
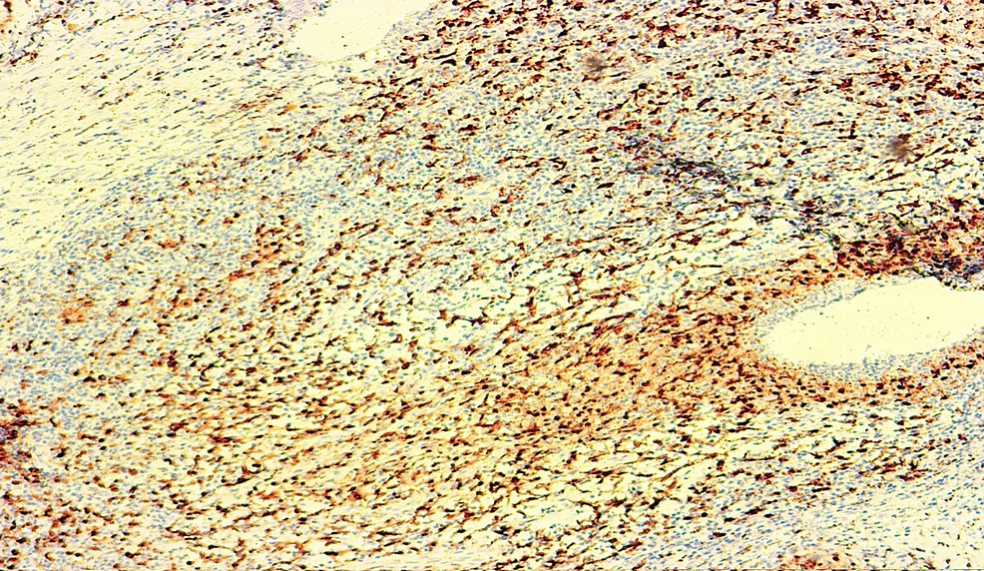
A photomicrograph, revealing the disseminated reactions in histiocytic cells (Immunohistochemistry, CD68; Original magnification, x100).

A 52-year-old Turkish woman visited our outpatient clinic with a tru-cut biopsy result of IGLM after undergoing different medical practices at several previous centers. On admission, her vital signs were within the normal limits. On the physical examination, a firm mass, approximately 20 mm in diameter was palpated in the upper inner quadrant of her left breast. The breast ultrasonography demonstrated a heterogeneous hypoechoic lesion, 20 mm in diameter with irregular contours and posterior acoustic shadowing in the upper inner quadrant of the left breast. The immunohistochemistry results for the excisional biopsy sample with wide local excision were as follows: granulomatous inflammation and positive reaction in the intact ducts for pan-cytokeratin; disseminated reactions in histiocytic cells for a cluster of differentiation 68 (CD68) (Figure 1); no myoepithelial cell loss in the intact ducts based on p63; no loss of myoepithelial layer based on smooth muscle actin (SMA) and Ki67; and focally positive immune reaction in the area of ​​hyperplasia based on Ki67. The histopathological evaluation in our vignette case revealed IGLM. The patient demonstrated an improved status and was discharged on hospital day 2 with additional medical treatment. No recurrence has occurred after the clinical and radiologic follow-up for four years and seven months. The patient was prescribed a course of medical peroral treatment to sustain the existing relief with a non-steroidal anti-inflammatory drug and a proton-pump inhibitor.

## Discussion

The aetiology of granulomatous mastitis has not been established, as various agents have been considered to date [3-5]. Various aetiologies have been postulated and propounded, such as tuberculosis, sarcoidosis, foreign body reactions, fungal and parasitic infections, and autoimmunity [6]. It occurs more commonly in women of childbearing age. Granulomatous mastitis is frequently observed in the third and fourth decades of life, but may occur at any age. Additionally, elderly individuals comprise an important age group among the male participants [3,7]. Recently, Martinez‐Ramos et al. reported a systematic review of 3060 patients, emphasizing the highest contribution of Mediterranean countries to the scientific literature on granulomatous mastitis in which Turkey plays a leading role [3].

Sonography and mammography findings exhibit a wide spectrum in IGLM, whereas dynamic contrast-enhanced magnetic resonance imaging has been counseled to enhance the specificity for the diagnostic purposes of IGLM [8]. In addition, C-reactive protein levels and neutrophil-to-lymphocyte ratio has been reported as non-beneficial tools, whereas white blood cells may act as a much more useful guide for the differential diagnosis of IGLM and breast abscesses [9].

Histopathologic examination is a necessary and gold-standard method for diagnosis, although B-mode ultrasonography provides crucial and specific findings, aiding in the identification of IGLM. Macroscopically, a firm, hard, and faintly nodular mass is recognized. Microscopically, granulomatous inflammation aggregating on lobules with lymphocytes, plasma cells, epithelioid histiocytes, multinucleated giant cells, and neutrophils are observed [10]. Interventional procedures have been utilized for diagnostic purposes. To this end, fine-needle aspiration (FNA) has been used historically. However, it is currently unpopular and is no longer supported due to its low sensitivity, which might be as low as 21%-39%, and difficulty in differentiating between IGLM and other granulomatous conditions of the breast. Finally, a core biopsy should be performed, following FNA to obtain accurate diagnosis [4,11].

A unilateral, firm, solitary breast mass with frequent inflammation of the overlying skin is the usual clinical presentation of IGLM, principally. Nevertheless, no ideal treatment for IGLM has been defined to date. Therefore, simple observation; drug therapies, such as steroids, antibiotics, corticosteroids, anti‐inflammatory drugs, immunosuppressive agents such as methotrexate and azathioprine; surgery, such as mastectomy and excision; and drainage have been proposed as the treatment modalities for this phenomenon. Antibiotics may be utilized when compatible with microbiological data available, with the chosen agents being directed against gram-positive organisms or in conjunction with surgical incision and drainage processes, particularly in cases wherein abscesses have developed. Corticosteroid therapy is frequently recommended for a period of three to six months. Posology is proposed for daily prednisone with doses as high as 60 mg with a gradual taper. Nevertheless, the long-term use of steroid therapy may lead to several side effects, such as glucose intolerance, weight gain, Cushing’s syndrome, hypertension, and steroid myopathy. Immunosuppressive agents such as methotrexate and azathioprine might be administrated both in order to avoid the long-term adverse effects of steroids and facilitate therapeutic purposes. Of note, wide local excision with or without steroid therapy has been proposed as the most commonly recommended approach [4,11-13].

Differential diagnoses include cystic neutrophilic granulomatous mastitis, CNGM, granulomatous mastitis due to Corynebacterium; other infections with granulomas, mycobacteria, fungi, cat scratch disease, and Brucella; granulomatosis with polyangiitis (Wegener's); sarcoidosis; squamous metaplasia of lactiferous ducts, SMOLD; and breast carcinoma in some challenging cases [10,14].

## Conclusions

This entity is a rare disorder with an unknown aetiology, and its diagnosis is difficult. It, per se, may easily imitate breast carcinoma based on its clinical and imaging findings, and there is no consensus for the most appropriate and definitive treatment for IGLM. Finally, we postulate that physicians should be vigilant and aware of this condition, so-called IGLM, to avoid overtreatment, unnecessary mastectomy, as if it was a malignancy, or overlooking an actual malignancy, as an aide memory. This issue merits further investigation. Additional studies are essential to address the specific and accurate diagnostic modalities that are optimal for resolving this issue.
